# Volume and quality of the gluteal muscles are associated with early physical function after total hip arthroplasty

**DOI:** 10.1007/s11548-025-03321-4

**Published:** 2025-01-21

**Authors:** Makoto Iwasa, Keisuke Uemura, Mazen Soufi, Yoshito Otake, Tomofumi Kinoshita, Tatsuhiko Kutsuna, Kazuma Takashima, Hidetoshi Hamada, Yoshinobu Sato, Nobuhiko Sugano, Seiji Okada, Masaki Takao

**Affiliations:** 1 Department of Orthopaedic Medical Engineering, Osaka University Graduate School of Medicine, 2-2, Yamadaoka, Suita, Osaka 565-0871 Japan; 2 Division of Information Science, Graduate School of Science and Technology, Nara Institute of Science and Technology, Ikoma, Japan; 3 Department of Orthopaedic Surgery, Ehime University Graduate School of Medicine, Matsuyama, Japan; 4 Department of Orthopaedic Surgery, Osaka University Graduate School of Medicine, Suita, Japan

**Keywords:** Artificial intelligence, Deep learning, Gluteal muscles, Muscle atrophy, Postoperative recovery, Timed up and go test

## Abstract

**Purpose:**

Identifying muscles linked to postoperative physical function can guide protocols to enhance early recovery following total hip arthroplasty (THA). This study aimed to evaluate the association of preoperative pelvic and thigh muscle volume and quality with early physical function after THA in patients with unilateral hip osteoarthritis (HOA).

**Methods:**

Preoperative Computed tomography (CT) images of 61 patients (eight males and 53 females) with HOA were analyzed. Six muscle groups were segmented from CT images, and muscle volume and quality were calculated on the healthy and affected sides. Muscle quality was quantified using the mean CT values (Hounsfield units [HU]). Early postoperative physical function was evaluated using the Timed Up & Go test (TUG) at three weeks after THA. The effect of preoperative muscle volume and quality of both sides on early postoperative physical function was assessed.

**Results:**

On the healthy and affected sides, mean muscle mass was 9.7 cm^3^/kg and 8.1 cm^3^/kg, and mean muscle HU values were 46.0 HU and 39.1 HU, respectively. Significant differences in muscle volume and quality were observed between the affected and healthy sides. On analyzing the function of various muscle groups, the TUG score showed a significant association with the gluteus maximum volume and the gluteus medius/minimus quality on the affected side.

**Conclusion:**

Patients with HOA showed significant muscle atrophy and fatty degeneration in the affected pelvic and thigh regions. The gluteus maximum volume and gluteus medius/minimus quality were associated with early postoperative physical function. Preoperative rehabilitation targeting the gluteal muscles on the affected side could potentially enhance recovery of physical function in the early postoperative period.

**Supplementary Information:**

The online version contains supplementary material available at 10.1007/s11548-025-03321-4.

## Introduction

Total hip arthroplasty (THA) is an effective treatment for end-stage hip osteoarthritis (HOA) offering benefits such as pain relief and improvement in range of motion and physical function [1]. Early recovery of physical function after THA surgery has been shown to reduce healthcare costs, facilitate early discharge from the hospital, and minimize social isolation. In contrast, a delay in recovery of physical function leads to higher healthcare costs and poor patient satisfaction [2]. Various attempts have been made to promote early functional recovery after THA, including surgical approaches, pain management, and rehabilitation protocols [3, 4]. For example, the anterior surgical approach has been shown to hasten postoperative physical functional recovery [5]. A systematic review showed that early implementation of rehabilitation after THA promotes physical functional recovery [6]. However, the optimal approach to preoperative rehabilitation is yet to be fully ascertained [4].

Identifying factors associated with post-THA physical function may clarify the focus of treatment and promote early recovery of physical function. Recent studies have aimed to identify factors associated with early postoperative physical function after THA based on preoperative grip strength and information collected using wearable sensors [7, 8]. Given the significant muscle atrophy commonly seen in the affected side of patients with HOA, combining preoperative and postoperative rehabilitation could potentially yield better recovery outcomes [10]. In addition, preoperative counseling about expected outcomes may help manage patients’ expectations and improve postoperative satisfaction [9]. Based on these studies, it is imperative to identify the factors associated with early postoperative physical function after THA.

Patients with HOA have significant atrophy and fatty degeneration of the pelvic and thigh muscles due to pain and difficulty in using the hip joint. While atrophy and fatty degeneration of the pelvic and thigh muscles are known to affect physical function [10], their effect on physical function in the early postoperative period is not well characterized. Identifying the muscles associated with postoperative physical function can help inform protocols to promote early recovery of the physical function postoperatively. Thus, the aim of this study was to identify the muscles of the pelvis and the thigh that affect early physical function after THA so that suitable treatment can be offered for each patient.

## Material and methods

### Participants

This was a retrospective study of prospectively collected data. All procedures involving human participants were performed following the ethical standards of the Institutional Research Committee (reference number: 11,321) and the 1964 Helsinki Declaration and its later amendments or comparable ethical standards. Initially, patients with unilateral HOA were selected from a cohort of 619 patients (105 males and 514 females) who underwent primary THA at our institution between November 2014 and 2020. Given that secondary HOA from developmental dysplasia of the hip is the most common reason for THA in Japan [11], the cohort included a high proportion of women. Patients were excluded if they had either bilateral hip disease, a history of pelvic or femoral trauma, infection, tumor, previous hip surgery, knee osteoarthritis, or lacked preoperative computed tomography (CT) scans. Following these exclusions, 125 patients with unilateral HOA were selected. Of these, 61 (8 males and 53 females) had preoperative and postoperative physical function data available for analysis. The mean (range) age, height, body weight, and body mass index were 62.3 ± 11.3 years (range 32–85), 156.3 ± 8.8 cm (range 143.0–184.0), 60.7 ± 14.3 kg (range 39.6–107.0), and 24.7 ± 5.0 kg/m^2^ (range 15.1–47.6), respectively. According to the Kellgren–Lawrence (KL) osteoarthritis grading system [12], the severity of HOA was grade III in nine patients and grade IV in 52 patients. The diagnosis of unilateral HOA was based on a preoperative radiograph and made by an orthopedic surgeon with 11 years of experience. Unilaterality was defined as asymptomatic healthy side and a joint space of at least 2 mm [13]. Asymptomatic hips were defined as those without pain, significant motion restriction, or functional limitations in daily activities, as assessed by patient interviews and radiological findings.

### Computed tomography imaging

Computed tomography images were acquired from the iliac crest to the femoral condyle using a standardized protocol (64-slice multislice Optima CT660 Pro; GE Healthcare Japan, Tokyo, Japan; 120 kV, 250 mA, helical pitch: 1.375:1, slice thickness: 1.25 mm, X-ray tube rotation speed: 0.6 s) [10]. All scans were performed with the patient in the supine position and the limb in a relaxed resting position to minimize the effect of muscle tension on measurements.

### Image analysis

**Fig. 1:**
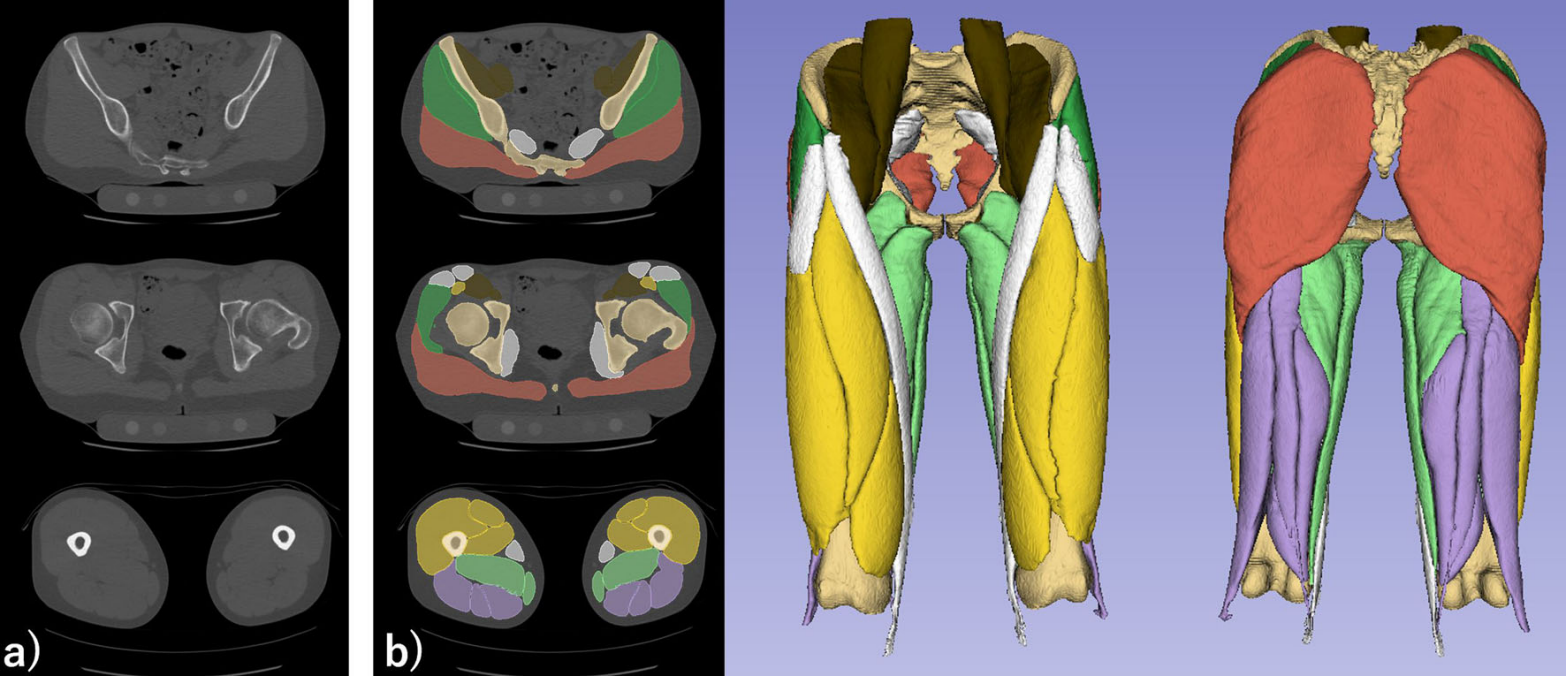
Three axial slices of a computed tomography image from a patient with left hip osteoarthritis (**a**), along with segmented images of muscle groups and bone regions, and the reconstructed 3D model (**b**). Muscle group color coding: Gluteus maximus (light brown), Gluteus medius and minimus (green), Iliopsoas (dark brown), Hip adductors (light green), Quadriceps (yellow), Hamstrings (purple), Other muscles (white)

The images were automatically extracted using the artificial intelligence system (Bayesian U-Net) previously developed by us [14]. Briefly, the model is based on the manual segmentation of the bone and the muscles. The system can perform segmentation of the muscles in a few minutes with a high accuracy (dice coefficient: 0.949). After the automated segmentation, an orthopedic surgeon (MI) specializing in musculoskeletal imaging reviewed and confirmed the accuracy of segmented muscles (Fig. 1b). This method has been used in a previous clinical study [10] assessing the correlation of muscle atrophy and fatty degeneration with health-related quality of life of patients with HOA.

The pelvic and thigh muscles were categorized into the following groups according to their function: the gluteus maximus; gluteus medius and minimus; iliopsoas (iliacus and psoas); hip adductors (pectineus, adductor major, adductor longus, adductor brevis, and gracilis); quadriceps (vastus lateralis, vastus medialis, vastus intermedius, and rectus femoris); and hamstrings (semitendinosus, semimembranosus, and biceps femoris).

Muscle volume was assessed bilaterally, normalized to body weight as in previous studies [15, 16]. Despite the relatively lean cohort, the body weight standardization ensured the broader applicability of the findings [15, 16]. Muscle quality was evaluated via CT values (Hounsfield unit [HU]), a validated metric for detecting fatty muscle infiltration [10, 17]. Comparisons were made between the affected and healthy sides to assess differences in muscle volume and quality.

### Assessment of postoperative physical function

Early postoperative physical function was assessed using the Timed Up & Go test (TUG), a widely recognized metric [18]. This test was conducted for 3 weeks post-THA, coinciding with hospital discharge and the resumption of basic daily activities, such as stair climbing [19]. Unlike studies assessing TUG performance a few days post-THA [2, 20], this study focused on a slightly later stage to evaluate movements relevant to social functioning [21] where a predictive relationship with future physical function is more apparent [22]. At 3 weeks postoperatively, patients typically exhibit some degree of recovery [23], allowing for the prediction of future physical function [24]. In Japan, this evaluation period is particularly clinically relevant as most patients are discharged to their homes and are independently capable of ascending and descending stairs by this time [19].

In the TUG, the patient is asked to get up from an armchair (45 cm high), walk to a cone three meters away, and then return to a sitting position in the chair. All patients wore shoes during the test and walked at their maximum possible speed. Patients were allowed to use walking aids if needed. TUG was performed four times and the average score was used for analysis.

The association between early postoperative physical function (i.e., TUG score) and muscle volume and muscle quality on the healthy and affected side was analyzed (termed here as “crude association analysis”). Further, as age and preoperative TUG score are known to be associated with post-THA physical function [2, 18], we investigated the association after adjusting for age and preoperative TUG score (termed here as “adjusted association analysis”).

To assess the impact of preoperative TUG scores, patients were divided into two groups based on the median preoperative TUG score. Patients with superior scores (i.e., lower TUG scores) were categorized as the “fast group,” whereas those with inferior scores (i.e., higher TUG scores) were classified as the “slow group.” The muscle volume and quality of the affected and healthy sides for each muscle group were compared between these two groups. Postoperative TUG scores were also evaluated for differences between the groups. In addition, the association between pain VAS and postoperative TUG scores at 3 weeks postoperatively was examined.

The influence of the walking method on postoperative TUG scores was assessed by analyzing data from 15 patients using a cane and 46 patients walking unaided at the time of the TUG test. Adjusted association analyses were conducted separately for patients using a cane and those walking unaided.

### Statistical analysis

The normality of continuous variables was assessed using the Shapiro–Wilk test. Paired Student’s *t*-test and Wilcoxon signed-rank tests evaluated differences between the affected and healthy sides. The association between early postoperative physical function and muscle volume and quality was analyzed using a two-step approach. A crude association analysis was conducted initially, followed by a multivariate ordered logistic regression analysis for adjusted associations. Postoperative TUG scores, used as a measure of postoperative physical function, were converted into ordinal variables based on quartile deviation [25]. Herein, postoperative TUG scores were categorized into five levels based on a quartile deviation of 2.7 s. The postoperative TUG score served as the dependent variable, whereas the volume and quality of preoperative muscle groups were the independent variables. In addition, the adjusted association analysis included age and preoperative TUG scores as independent variables. To assess the impact of the walking method on postoperative TUG scores, coefficients were calculated using the levels of the postoperative TUG score as the dependent variable. Furthermore, adjusted association analyses were performed using ordinal logistic regression analysis to examine the association between postoperative TUG score rank and cane use in patients walking with a cane versus those walking unaided.

The correlation between postoperative pain, measured using a VAS, and TUG scores was evaluated using Spearman’s rank correlation coefficient. Statistical analyses were conducted using JMP® 15 (SAS Institute Inc., Cary, NC, USA). A *p* value < 0.05 was considered statistically significant. The required sample size was calculated using G*Power version 3.1.9.6 [26], approximating the probabilities for one category transition (Pr(Y = 1/X = 1) H1 = 0.46 and Pr(Y = 1/X = 1) H0 = 0.64), with an α error of 0.05 and a power of 0.80.

## Results

### Muscle volume and quality

On the healthy side, the mean volume of each muscle was 9.7 cm^3^/kg (range 2.9–18.7), and the mean quality of each muscle was 46.0 HU (range 33.5–57.3). On the affected side, the total mean muscle volume was 8.1 cm^3^/kg (range 2.2–16.0), and the mean muscle quality was 39.1 HU (range 22.1–51.2). The comparison of the muscle groups of the affected and healthy sides revealed significant atrophy and fatty degeneration in all muscle groups on the affected side (Table 1).

**Table 1:** Comparison of the volume and quality of the pelvic and thigh muscles on the affected side and the healthy side

		Healthy side	Affected side	*p* value
Volume (cm^3^/kg)	Gluteus maximus	11.8 (7.2–17.8)	9.6 (5.1–14.1)	< 0.01^*1^
	Gluteus medius and minimus	5.8 (3.8–8.8)	5.0 (1.9–7.9)	< 0.01^*1^
	Iliopsoas	2.9 (1.8–4.6)	2.2 (1.0–3.8)	< 0.01^*1^
	Hip adductors	11.2 (6.2–17.8)	9.0 (5.3–14.8)	< 0.01^*1^
	Quadriceps	18.7 (7.2–29.5)	16.0 (6.7–25.8)	< 0.01^*1^
	Hamstrings	7.7 (4.1–10.1)	6.7 (4.0–9.8)	< 0.01^*1^
Quality (HU)	Gluteus maximus	33.5 (7.7–50.9)	22.2 (−7.8 to 45.2)	< 0.01^*1^
	Gluteus medius and minimus	45.0 (21.5–58.2)	34.6 (−6.1 to 54.8)	< 0.01^*^^2^
	Iliopsoas	57.3 (43.1–65.9)	51.2 (25.7–66.1)	< 0.01*^2^
	Hip adductors	45.0 (25.5–52.7)	38.5 (16.6–52.2)	< 0.01*^2^
	Quadriceps	53.5 (29.8–62.2)	50.8 (31.0–63.0)	0.02*^2^
	Hamstrings	41.7 (20.7–52.3)	37.0 (13.3–54.0)	< 0.01*^2^

*HU* Hounsfield unit. Data expressed as mean (range)

^*1^Significantly different between the sides (paired student’s *t*-test)

^*2^Significantly different between the sides (Wilcoxon signed-rank test)

### Physical function and pain

The median preoperative TUG score was 8.8 s (range 5.3–29.5 s), whereas the median postoperative TUG score was 9.0 s (range 6.4–21.5), with no significant difference (*p* = 0.21). Among subgroups, the median postoperative TUG score was 8.6 s (range 6.4–15.1) in the fast group and 10.3 s (range 7.3–21.5) in the slow group, showing a significant difference (*p* < 0.01). The volume and quality of each muscle group on the affected and healthy sides, categorized by the fast and slow groups, are detailed in Supplementary Table 1. The median postoperative pain VAS score was 8.0 (range 0–92). No significant correlation was observed between the postoperative TUG score and postoperative pain VAS (*ρ* = 0.191, *p* = 0.141).

### Association of TUG with muscle volume and quality (crude association analysis)

On the healthy side, early postoperative physical function was significantly associated with HU values for all muscles except the gluteus maximus, while volume was significantly associated only with the quadriceps (Table 2). On the affected side, HU values for all muscles and volume for all muscles except the iliopsoas were significantly associated with early postoperative physical function (Table 2).

**Table 2:** Association of early postoperative physical function with muscle volume and quality

			*β*	SE	95% CI	*p* value
Healthy side	Volume (cm^3^/kg)	Gluteus maximus	− 0.14	0.15	− 0.45 to 0.15	0.34
		Gluteus medius and minimus	− 0.36	0.26	− 0.88 to 0.14	0.15
		Iliopsoas	− 0.15	0.43	− 0.99 to 0.68	0.71
		Hip adductors	− 0.13	0.14	− 0.40 to 0.14	0.34
		Quadriceps	− 0.16	0.07	− 0.31 to − 0.01	0.03*
		Hamstrings	− 0.26	0.21	− 0.68 to 0.15	0.21
	Quality (HU)	Gluteus maximus	− 0.05	0.02	− 0.10 to 0.01	0.06
		Gluteus medius and minimus	− 0.09	0.03	− 0.16 to − 0.03	< 0.01*
		Iliopsoas	− 0.14	0.05	− 0.26 to − 0.03	< 0.01*
		Hip adductors	− 0.10	0.04	− 0.19 to − 0.01	0.03*
		Quadriceps	− 0.10	0.04	− 0.18 to − 0.01	0.02*
		Hamstrings	− 0.08	0.03	− 0.14 to − 0.01	0.01*
Affected side	Volume (cm^3^/kg)	Gluteus maximus	− 0.39	0.15	− 0.70 to − 0.08	< 0.01*
		Gluteus medius and minimus	− 0.52	0.25	− 1.03 to − 0.02	0.04*
		Iliopsoas	− 0.48	0.38	− 1.23 to 0.26	0.20
		Hip adductors	− 0.25	0.12	− 0.50 to − 0.01	0.04*
		Quadriceps	− 0.23	0.08	− 0.41 to − 0.06	< 0.01*
		Hamstrings	− 0.44	0.21	− 0.86 to − 0.03	0.03*
	Quality (HU)	Gluteus maximus	− 0.05	0.02	− 0.09 to − 0.01	< 0.01*
		Gluteus medius and minimus	− 0.06	0.02	− 0.10 to − 0.02	< 0.01*
		Iliopsoas	− 0.06	0.03	− 0.12 to − 0.01	0.02*
		Hip adductors	− 0.07	0.03	− 0.13 to − 0.01	0.01*
		Quadriceps	− 0.12	0.04	− 0.20 to − 0.04	< 0.01*
		Hamstrings	− 0.06	0.02	− 0.11 to − 0.01	0.02*

95% CI, 95% confidence interval; *β*, standard regression coefficient; HU, Hounsfield unit; *SE*, standard error

*Significant association (ordinal logistic regression analysis)

### Association of TUG with muscle volume and quality (adjusted association analysis)

After adjusting for age and preoperative TUG score (i.e., adjusted association analysis), the volume of the gluteus maximus and the quality of the gluteus medius and minimus on the affected side remained significant (Table 3).

**Table 3:** Association of early postoperative physical function with muscle volume and quality adjusted for age and preoperative TUG score

			*β*	*SE*	95% CI	*p* value
Healthy side	Volume (cm^3^/kg)	Gluteus maximus	− 0.09	0.16	− 0.42 to 0.22	0.16
		Gluteus medius and minimus	− 0.17	0.27	− 0.70 to 0.35	0.52
		Iliopsoas	0.06	0.46	− 0.84 to 0.96	0.89
		Hip adductors	− 0.08	0.15	− 0.38 to 0.20	0.56
		Quadriceps	− 0.10	0.08	− 0.27 to 0.06	0.22
		Hamstrings	− 0.11	0.23	− 0.56 to 0.34	0.63
	Quality (HU)	Gluteus maximus	− 0.04	0.03	− 0.11 to 0.01	0.14
		Gluteus medius and minimus	− 0.04	0.04	− 0.12 to 0.03	0.31
		Iliopsoas	− 0.07	0.06	− 0.21 to 0.05	0.24
		Hip adductors	− 0.06	0.05	− 0.16 to 0.03	0.18
		Quadriceps	− 0.06	0.04	− 0.16 to 0.02	0.13
		Hamstrings	− 0.05	0.04	− 0.13 to 0.02	0.15
Affected side	Volume (cm^3^/kg)	Gluteus maximus	− 0.36	0.16	− 0.69 to − 0.03	0.03*
		Gluteus medius and minimus	− 0.46	0.27	− 1.00 to to 0.07	0.09
		Iliopsoas	− 0.17	0.41	− 0.97 to 0.63	0.67
		Hip adductors	− 0.17	0.13	− 0.44 to 0.09	0.19
		Quadriceps	− 0.16	0.09	− 0.35 to 0.03	0.09
		Hamstrings	− 0.33	0.23	− 0.78 to 0.12	0.15
	Quality (HU)	Gluteus maximus	− 0.04	0.02	− 0.09 to 0.01	0.06
		Gluteus medius and minimus	− 0.04	0.02	− 0.09 to − 0.01	0.04*
		Iliopsoas	− 0.04	0.03	− 0.11 to 0.02	0.20
		Hip adductors	− 0.04	0.03	− 0.11 to 0.02	0.21
		Quadriceps	− 0.07	0.04	− 0.17 to 0.01	0.10
		Hamstrings	− 0.02	0.03	− 0.09 to 0.03	0.40

95% CI, 95% confidence interval; *β*, standard regression coefficient; HU, Hounsfield unit; *SE*, standard error

*Significant association (ordinal logistic regression analysis)

### Effect of cane use on postoperative TUG scores and adjusted association analysis

The walking method (unaided walking vs. walking with a cane) did not considerably influence postoperative TUG scores (coefficient =  − 1.02, standard error = 0.55, 95% confidence interval: − 2.11–0.07, *p* = 0.07). Adjusted association analyses revealed significant associations for gluteus maximus volume in unaided walkers and for gluteus medius and minimus quality in patients using a cane (Supplementary Tables 2 and 3).

## Discussion

To the best of our knowledge, this is the first study to investigate the association between preoperative pelvic and thigh muscles and early postoperative physical function after THA in patients with HOA. We found significant atrophy and fatty degeneration in the pelvic and thigh muscles of the affected side, most of which were associated with early postoperative physical function. In particular, the volume of gluteus maximus and the quality of gluteus medius and gluteus minimus on the affected side showed a strong association with early postoperative physical function. These findings suggest that rehabilitation interventions for these muscles may improve early postoperative physical function. These differences in volume and quality may be attributed to the varying proportions of muscle fiber types in the gluteus maximus compared with the gluteus medius and minimus [27, 28]. Studies have demonstrated differences in the progression of atrophy and degeneration between muscle fiber types [29]. Although training is expected to be effective for both muscle fiber types [30, 31], further research is required to determine the optimal rehabilitation protocol for enhancing muscle volume and quality.

### Association of postoperative physical function with muscle volume and quality

Some previous studies have assessed the association between early postoperative physical function and muscle volume and quality on the operated and healthy side. For instance, in the study by Ohmori et al., the postoperative walking speed of HOA patients was associated with muscle strength of the healthy side, indicating the importance of the muscles of the healthy side [32]. However, some studies have found the importance of the affected side muscles on postoperative function. For instance, in the study by Holstege et al., the preoperative knee extensor strength of the affected side was associated with the Western Ontario and McMaster Universities Arthritis Index Physical Function score at 12 weeks after THA [33]. The findings of our study may support reports suggesting preoperative intervention for the affected side. However, a direct comparison with the previous reports cannot be made because of differences regarding the method/parameter used to quantify physical function. Specifically, physical function is often performed using patient-reported outcome measures, and its usefulness has been reported [34]. However, patient-reported outcome measures are liable to overestimation due to postoperative analgesia [35] and underestimation due to postoperative anxiety [36]. This study assessed physical function with the TUG score, which can comprehensively evaluate hip function [2, 18]. Specifically, the TUG combines daily activities such as getting up from a chair, walking, turning a cone, and sitting activities; therefore, muscle strength of the lower extremities can be evaluated through walking and sit-to-stand activities, and balance control can be evaluated through changing direction [32, 37]. As complex activities similar to the TUG are required in daily living, postoperative physical function evaluated using the TUG is likely more clinically relevant than other methods.

### Muscle parameters affecting the postoperative physical function after adjusting for age and preoperative TUG score

In the present study, the volume of the gluteus maximus and the quality of the gluteus minimus and medius were associated with TUG score after adjusting for age and preoperative TUG score. This is consistent with previous studies demonstrating the importance of the gluteal muscles on physical function. The gluteus maximus, a hip extensor, produces forward propulsion and is associated with walking speed [38]. Further, it also serves as a hip abductor and is associated with postural stability, including braking during walking and with standing and sitting movements [39, 40]. Based on this information, Ukai et al. investigated muscle strength in hip flexion, extension, and abduction after THA and confirmed preoperative gluteus maximus volume as an important indicator of post-THA physical function [41].

Studies have shown that gluteus medius and minimus play an essential role in maintaining pelvic stability during exercise and contribute to postural stability during activities of daily living [42]. For instance, studies have reported that the degree of fatty degeneration in the gluteus medius and minimus is related to the risk of falls and fractures in the elderly. Collectively, the results of the present study support the previous reports on the importance of the gluteal muscles on physical function.

### Relationship between postoperative physical function, preoperative conditions, and pain

Patients with superior preoperative TUG scores achieved better postoperative TUG outcomes, consistent with the findings of an association between pre- and mid-postoperative TUG scores (6 months) [18]. Comparisons between the fast and slow groups revealed considerable differences in most muscle groups on the healthy side (Supplementary Table 1). However, only about half of the affected-side muscles showed substantial differences (Supplementary Table 1). This suggests that muscle atrophy on the healthy side in patients with end-stage HOA contributes to functional decline [43]. Preoperative physical function may thus depend on the remaining functional capacity of the healthy side.

Postoperative pain scores varied among patients but showed no substantial association with postoperative TUG scores, aligning with findings by Winther et al. [44], who reported no impact of pain on training load or muscle performance in patients with postoperative THA. These results suggest that postoperative pain had minimal influence on the findings of this study.

### Effect of cane use on postoperative TUG scores

Previous studies have not analyzed the impact of cane use on postoperative TUG scores [2, 20, 34]. Our study revealed that the gluteus maximus primarily influenced TUG scores in unaided walkers, whereas the gluteus medius and minimus played a more substantial role in patients who were cane assisted. Atrophy of the gluteus medius and minimus, which are vital for pelvic stabilization during directional changes [42], likely contributed to delays in direction changes among cane users during the TUG [45]. Conversely, the gluteus maximus, affecting walking speed [38], influenced TUG scores in patients walking unaided. As the TUG evaluates a series of movements—such as standing up, walking, turning, and sitting down—further research could analyze the time required for each component to provide a more detailed functional assessment.

### Limitations

Some limitations of this study should be acknowledged. First, the relatively small sample size precluded an evaluation of the combined effects of muscle volume and quality on early postoperative physical function. While sub-analyses, such as the impact of pain and cane use, were conducted, larger studies are needed to identify additional factors influencing physical function. Second, most participants were women with lower BMI, limiting the generalizability of findings to men and individuals with higher BMI. Given evidence of sex-based differences in muscle function during exercise [46], further research is warranted. Finally, muscles were categorized by function and were evaluated in groups; individual muscles were not examined. TUG used to assess physical function is a complex movement, and clarifying the relationship between individual muscles and movements may help optimize treatment strategies.

## Conclusion

In this study, the volume of gluteus maximum and the quality of gluteus medius/minimus quality on the affected side were associated with postoperative physical function evaluated using the TUG test at three weeks after THA in HOA patients. Our results suggest that preoperative training focusing on the gluteus muscles of the affected side may lead to early recovery of physical function after THA.

## Supplementary Information

Below is the link to the electronic supplementary material.

Supplementary file1 (DOCX 20 kb)

Supplementary file2 (DOCX 21 kb)

Supplementary file3 (DOCX 21 kb)

## Data Availability

The data that support the findings of this study are available from the corresponding author upon reasonable request.
