# Supplementary material for: Volume and quality of the gluteal muscles are associated with early physical function after total hip arthroplasty
Source: Int J Comput Assist Radiol Surg. 2025 Jan 21;20(4):703–11. doi: 10.1007/s11548-025-03321-4 (PMC12034588; doi:10.1007/s11548-025-03321-4)
Supplement: Supplementary file 1 — Supplementary file1 (DOCX 20 kb) [file 11548_2025_3321_MOESM1_ESM.docx]

**Supplementary Table 1.** Comparison of the volume and quality of the pelvic and thigh muscles in the fast group and the slow group

|  |  |  | Fast group | Slow group | *p* value |
| --- | --- | --- | --- | --- | --- |
| Healthy side | **Volume (cm^3^/kg)** | Gluteus maximus | 12.8 (9.6 – 17.8) | 10.9 (7.2 – 14.3) | <0.01^*^ |
|  |  | Gluteus medius and minimus | 5.9 (4.3 – 8.8) | 5.4 (3.8 – 7.5) | 0.04^*^ |
|  |  | Iliopsoas | 3.0 (2.2 – 4.6) | 2.6 (1.8 – 3.7) | 0.03^*^ |
|  |  | Hip adductors | 11.5 (8.6 – 17.8) | 10.8 (6.2 – 14.7) | 0.18 |
|  |  | Quadriceps | 19.8 (14.9 – 29.5) | 17.1 (7.2 – 24.0) | <0.01^*^ |
|  |  | Hamstrings | 8.1 (6.0 – 10.1) | 7.5 (4.1 – 9.6) | <0.01^*^ |
|  | **Quality (HU)** | Gluteus maximus | 37.2 (7.7 – 50.9) | 30.9 (13.6 – 44.0) | <0.01^*^ |
|  |  | Gluteus medius and minimus | 50.5 (34.6 – 58.2) | 43.5 (21.5 – 54.8) | <0.01^*^ |
|  |  | Iliopsoas | 59.2 (49.1 – 65.9) | 56.9 (43.1 – 64.1) | 0.01^*^ |
|  |  | Hip adductors | 47.0 (32.6 – 52.7) | 43.8 (25.5 – 51.8) | 0.03^*^ |
|  |  | Quadriceps | 54.6 (42.4 – 62.2) | 53.3 (29.8 – 59.8) | 0.04^*^ |
|  |  | Hamstrings | 44.3 (25.9 – 52.3) | 40.5 (20.7 – 51.5) | 0.04^*^ |
| Affected side | **Volume (cm^3^/kg)** | Gluteus maximus | 9.7 (6.2 – 14.1) | 9.2 (5.1 – 13.6) | 0.13 |
|  |  | Gluteus medius and minimus | 5.0 (3.1 – 7.9) | 4.7 (1.9 – 6.5) | 0.43 |
|  |  | Iliopsoas | 2.3 (1.2 – 3.8) | 2.1 (1.0 – 3.3) | 0.44 |
|  |  | Hip adductors | 9.9 (5.9 – 14.8) | 8.3 (5.3 – 12.4) | 0.10 |
|  |  | Quadriceps | 17.1 (12.3 – 25.8) | 14.9 (6.7 – 20.0) | <0.01^*^ |
|  |  | Hamstrings | 6.8 (4.8 – 9.8) | 6.2 (4.0 – 8.4) | <0.01^*^ |
|  | **Quality (HU)** | Gluteus maximus | 25.5 (-2.6 – 45.2) | 22.6 (-7.8 – 39.6) | 0.03^*^ |
|  |  | Gluteus medius and minimus | 40.8 (21.4 – 52.6) | 32.8 (-6.1 – 54.8) | 0.02^*^ |
|  |  | Iliopsoas | 53.7 (34.6 – 63.4) | 52.9 (25.7 – 66.1) | <0.01^*^ |
|  |  | Hip adductors | 42.0 (27.0 – 52.2) | 38.0 (16.6 – 49.7) | 0.07 |
|  |  | Quadriceps | 53.1 (40.4 – 61.2) | 49.5 (31.0 – 63.0) | 0.01^*^ |
|  |  | Hamstrings | 40.2 (22.1 – 54.0) | 34.0 (13.3 – 51.4) | 0.03^*^ |

HU, Hounsfield unit. Data expressed as mean (range).

^*^Significantly different between the groups (Wilcoxon signed-rank test).
