# Supplementary material for: Volume and quality of the gluteal muscles are associated with early physical function after total hip arthroplasty
Source: Int J Comput Assist Radiol Surg. 2025 Jan 21;20(4):703–11. doi: 10.1007/s11548-025-03321-4 (PMC12034588; doi:10.1007/s11548-025-03321-4)
Supplement: Supplementary file 2 — Supplementary file2 (DOCX 21 kb) [file 11548_2025_3321_MOESM2_ESM.docx]

**Supplementary Table 2.** Association of early postoperative physical function with muscle volume and quality adjusted for age and preoperative TUG score in patients walking unaided

|  |  |  | *β* | SE | 95% CI | *p* value |
| --- | --- | --- | --- | --- | --- | --- |
| Healthy side | **Volume (cm^3^/kg)** | Gluteus maximus | -0.18 | 0.19 | -0.56 – 0.19 | 0.34 |
|  |  | Gluteus medius and minimus | -0.17 | 0.31 | -0.77 – 0.43 | 0.58 |
|  |  | Iliopsoas | 0.13 | 0.53 | -0.90 – 1.17 | 0.80 |
|  |  | Hip adductors | -0.09 | 0.16 | -0.42 – 0.22 | 0.54 |
|  |  | Quadriceps | -0.11 | 0.09 | -0.30 – 0.07 | 0.25 |
|  |  | Hamstrings | -0.23 | 0.26 | -0.75 – 0.28 | 0.37 |
|  | **Quality (HU)** | Gluteus maximus | 0.01 | 0.03 | -0.06 – 0.08 | 0.77 |
|  |  | Gluteus medius and minimus | -0.06 | 0.05 | -0.17 – -0.03 | 0.21 |
|  |  | Iliopsoas | -0.07 | 0.07 | -0.22 – 0.08 | 0.35 |
|  |  | Hip adductors | -0.06 | 0.06 | -0.19 – 0.07 | 0.36 |
|  |  | Quadriceps | -0.10 | 0.06 | -0.22 – 0.02 | 0.12 |
|  |  | Hamstrings | -0.03 | 0.05 | -0.13 – 0.06 | 0.47 |
| Affected side | **Volume (cm^3^/kg)** | Gluteus maximus | -0.46 | 0.21 | -0.88 – -0.04 | 0.03^*^ |
|  |  | Gluteus medius and minimus | -0.26 | 0.30 | -0.86 – 0.33 | 0.38 |
|  |  | Iliopsoas | -0.09 | 0.49 | -1.05 – 0.86 | 0.84 |
|  |  | Hip adductors | -0.18 | 0.15 | -0.48 – 0.12 | 0.24 |
|  |  | Quadriceps | -0.14 | 0.12 | -0.38 – 0.09 | 0.22 |
|  |  | Hamstrings | -0.30 | 0.26 | -0.82 – 0.22 | 0.25 |
|  | **Quality (HU)** | Gluteus maximus | -0.02 | 0.02 | -0.07 – 0.03 | 0.41 |
|  |  | Gluteus medius and minimus | -0.03 | 0.03 | -0.10 – 0.03 | 0.30 |
|  |  | Iliopsoas | -0.02 | 0.04 | -0.12 – 0.07 | 0.59 |
|  |  | Hip adductors | -0.02 | 0.05 | -0.12 – 0.07 | 0.59 |
|  |  | Quadriceps | -0.08 | 0.06 | -0.21 – 0.03 | 0.16 |
|  |  | Hamstrings | -0.01 | 0.03 | -0.08 – 0.06 | 0.77 |

95% CI, 95% confidence interval; β, standard regression coefficient; HU, Hounsfield unit; SE, standard error.

*Significant association (ordinal logistic regression analysis).
