# Supplementary material for: Volume and quality of the gluteal muscles are associated with early physical function after total hip arthroplasty
Source: Int J Comput Assist Radiol Surg. 2025 Jan 21;20(4):703–11. doi: 10.1007/s11548-025-03321-4 (PMC12034588; doi:10.1007/s11548-025-03321-4)
Supplement: Supplementary file 3 — Supplementary file3 (DOCX 21 kb) [file 11548_2025_3321_MOESM3_ESM.docx]

**Supplementary Table 3.** Association of early postoperative physical function with muscle volume and quality adjusted for age and preoperative TUG score in ambulatory patients in patients walking with a cane

|  |  |  | *β* | SE | 95% CI | *p* value |
| --- | --- | --- | --- | --- | --- | --- |
| Healthy side | **Volume (cm^3^/kg)** | Gluteus maximus | 0.31 | 0.43 | -0.54 – 1.16 | 0.47 |
|  |  | Gluteus medius and minimus | -0.21 | 0.70 | -1.59 – 1.15 | 0.75 |
|  |  | Iliopsoas | 0.71 | 1.25 | -1.74 – 3.16 | 0.57 |
|  |  | Hip adductors | 0.31 | 0.62 | -0.91 – 1.53 | 0.61 |
|  |  | Quadriceps | 0.02 | 0.29 | -0.55 – 0.61 | 0.92 |
|  |  | Hamstrings | 0.24 | 0.52 | -0.77 – 1.27 | 0.63 |
|  | **Quality (HU)** | Gluteus maximus | -0.08 | 0.07 | -0.22 – 0.05 | 0.24 |
|  |  | Gluteus medius and minimus | -0.08 | 0.08 | -0.24 – 0.07 | 0.28 |
|  |  | Iliopsoas | -0.23 | 0.15 | -0.53 – 0.07 | 0.13 |
|  |  | Hip adductors | -0.04 | 0.07 | -0.19 – 0.10 | 0.54 |
|  |  | Quadriceps | -0.05 | 0.07 | -0.19 – 0.07 | 0.40 |
|  |  | Hamstrings | -0.07 | 0.07 | -0.22 – 0.07 | 0.34 |
| Affected side | **Volume (cm^3^/kg)** | Gluteus maximus | -0.31 | 0.35 | -1.00 – 0.37 | 0.36 |
|  |  | Gluteus medius and minimus | -0.35 | 0.64 | -1.62 – 0.90 | 0.58 |
|  |  | Iliopsoas | -0.84 | 0.90 | -2.61 – 0.92 | 0.35 |
|  |  | Hip adductors | -0.26 | 0.32 | -0.91 – 0.37 | 0.41 |
|  |  | Quadriceps | -0.30 | 0.24 | -0.78 – 0.18 | 0.21 |
|  |  | Hamstrings | -0.11 | 0.49 | -1.09 – 0.85 | 0.81 |
|  | **Quality (HU)** | Gluteus maximus | -0.06 | 0.05 | -0.08 – 0.12 | 0.67 |
|  |  | Gluteus medius and minimus | -0.07 | 0.04 | -0.15 – -0.001 | 0.04^*^ |
|  |  | Iliopsoas | -0.06 | 0.05 | -0.16 – 0.04 | 0.23 |
|  |  | Hip adductors | -0.06 | 0.06 | -0.18 – 0.05 | 0.27 |
|  |  | Quadriceps | -0.06 | 0.08 | -0.23 – 0.10 | 0.42 |
|  |  | Hamstrings | -0.05 | 0.07 | -0.21 – 0.09 | 0.45 |

95% CI, 95% confidence interval; *β*, standard regression coefficient; HU, Hounsfield unit; *SE*, standard error.

^*^Significant association (ordinal logistic regression analysis).
